# Bovine *In Vitro* Oocyte Maturation and Embryo Production Used as a Model for Testing Endocrine Disrupting Chemicals Eliciting Female Reproductive Toxicity With Diethylstilbestrol as a Showcase Compound

**DOI:** 10.3389/ftox.2022.811285

**Published:** 2022-05-24

**Authors:** K. Asimaki, P. Vazakidou, H. T. A. van Tol, C. H. Y. Oei, E. A. Modder, M. B. M. van Duursen, B. M. Gadella

**Affiliations:** ^1^ Division of Farm Animal Health, Department Population Health Sciences, Faculty of Veterinary Medicine, Utrecht University, Utrecht, Netherlands; ^2^ Amsterdam Institute for Life and Environment, Section Environment and Health, Vrije Universiteit Amsterdam, Amsterdam, Netherlands

**Keywords:** diethylstilbestrol, oocyte maturation, cumulus expansion, steroidogenesis, blastocyst, *in vitro* model, bovine

## Abstract

Endocrine disrupting chemicals (EDCs) can interfere with normal hormonal action and regulation. Exposure of women to EDCs has been associated with adverse reproductive health outcomes. The assays currently used to identify EDCs that elicit female reproductive toxicity lack screening tests that address effects on the maturation of oocytes, a process that enables them to be fertilized and develop into embryos. Here, a screening method employing the bovine model of *in vitro* oocyte maturation and embryo production is described. Endpoints explored address important events in oocyte maturation and developmental competence acquisition. To test the method, the effects of the known human EDC diethylstilbestrol (DES; an estrogen receptor agonist) were evaluated in a range of concentrations (10^–9^ M, 10^–7^ M, 10^–5^ M). Bovine oocytes were exposed to DES during *in vitro* maturation (IVM) or embryos were exposed during *in vitro* embryo culture (IVC). The endpoints evaluated included nuclear maturation, mitochondrial redistribution, cumulus cell expansion, apoptosis, and steroidogenesis. DES-exposed oocytes were fertilized to record embryo cleavage and blastocyst rates to uncover effects on developmental competence. Similarly, the development of embryos exposed to DES during IVC was monitored to assess the impact on early embryo development. Exposure to 10^–9^ M or 10^–7^ M DES did not affect the endpoints addressing oocyte maturation or embryo development. However, there were considerable detrimental effects observed in oocytes exposed to 10^–5^ M DES. Specifically, compared to vehicle-treated oocytes, there was a statistically significant reduction in nuclear maturation (3% vs 84%), cumulus expansion (2.8-fold vs 3.6-fold) and blastocyst rate (3% vs 32%). Additionally, progesterone and pregnenolone concentrations measured in IVM culture media were increased. The screening method described here shows that bovine oocytes were sensitive to the action of this particular chemical (i.e., DES), albeit at high concentrations. In principle, this method provides a valuable tool to assess the oocyte maturation process and early embryo development that can be used for reproductive toxicity screening and possibly EDC identification. Further studies should include EDCs with different mechanisms of action and additional endpoints to further demonstrate the applicability of the bovine oocyte model for chemical risk assessment purposes and EDC identification.

## 1 Introduction

The production and use of chemicals have increased tremendously over the past years, and will continue to increase, to accommodate the high living standards in modern society (UNEP, 2019). Unfortunately, many of these chemicals can be harmful to the environment and human health (Landrigan et al., 2018). Of particular concern are the endocrine disrupting chemicals (EDC). EDCs are defined as exogenous substances or mixtures that alter the endocrine system and consequently cause adverse health effects to an intact organism, its progeny or (sub)populations (International Programme on Chemical Safety, 2002). A classic example of an EDC eliciting adverse health effects in humans, is the synthetic estrogen receptor agonist diethylstilbestrol (DES), which has been shown to cause aberrations in reproductive processes in women and their *in utero* exposed progeny (Newbold et al., 2006). DES can bind and activate nuclear estrogen receptors (ER) and thus can modulate estrogen dependent gene transcription (Nikov et al., 2001; Adam et al., 2020). Studies suggest that non-genomic effects directed by intracellular cell signaling can also occur, e.g., through activation of transmembrane estrogen receptors (Nadal et al., 2000; Alonso-Magdalena et al., 2005; Tokumoto et al., 2007; Zhang et al., 2014). The physiological ER agonist, 17-βestradiol (E2), constitutes an important link of the hypothalamic-pituitary-ovarian axis contributing to the regulation of gonadotrophin secretion that governs ovarian cyclicity. Within the adult ovary, estrogens have a less well characterized role, but it is beyond a doubt that estrogens contribute to the development of the functional unit of the ovary, the follicle (Emmen et al., 2005; Dewailly et al., 2016; Jolivet et al., 2021).

Female reproductive health, as evidenced by the example of DES, may be affected by exposure to EDCs. Oocyte cytoplasmic and meiotic maturation that confer to the oocyte the ability to be successfully fertilized and to support early embryonic development to the blastocyst stage (developmental competence) are important factors contributing to female reproductive health (Conti and Franciosi, 2018). These processes are tightly bound to the spatiotemporal control of hormone production and action, and therefore potentially susceptible to EDC action. Indeed, certain EDCs have been reported to impede aspects of oocyte maturation and developmental competence acquisition, as shown by *in vitro* studies in different animal models (Mlynarcíková et al., 2009; Petro et al., 2012a; Patel et al., 2015). Despite the concern regarding EDC effects on female reproduction, there is a lack of adequate test methods to assess potential interaction with oocyte maturation and early embryo development. This gap in test capabilities poses a threat for reproductive health world-wide. A promising method includes the identification of EDCs eliciting reproductive toxicity with an assay employing the bovine model of *in vitro* oocyte maturation and embryo production. The applicability of this model has been previously demonstrated in the context of reproductive toxicology by Luciano et al. (2010) and Beker van Woudenberg et al. (2012). Oocyte maturation and embryo production in humans and in the bovine have been reported to share similarities, thereby strongly supporting the applicability of this model for toxicological studies (Ménézo and Hérubel, 2002; Santos et al., 2014). Importantly, the biological material required, i.e., oocytes isolated from ovaries dissected post-mortem from slaughterhouse cows, allows for compliance with the 3R principle. The evidence pointing towards a disruption of normal reproductive function by EDCs is constantly growing and, considering the wide exposure of humans to these chemicals, there is a pressing need to develop effective strategies for their identification and the regulation of their usage.

Here, we present the bovine model of *in vitro* oocyte maturation and embryo production in an effort to pinpoint endpoints sensitive to EDC action. This model includes the *in vitro* maturation (IVM) of cumulus-oocyte complexes in a defined medium, *in vitro* fertilization by co-incubation with sperm, and subsequent *in vitro* embryo culture (IVC) of the presumptive zygotes for 8 days, until the blastocyst stage. To test the model, we have used DES as a showcase compound due to its established and well documented action as a human-relevant endocrine disruptor. Oocytes were exposed to DES during IVM, or embryos were exposed to DES during IVC. The endpoints selected to be evaluated represent important aspects of oocyte maturation and embryo development. These include oocyte nuclear maturation, oocyte mitochondrial redistribution, cumulus cell expansion, oocyte and cumulus cell apoptosis, steroid hormone production, and *in vitro* embryo development monitored by cleavage and blastocyst rate (see also Table 1). The biological importance of these endpoints for reproductive health, and therefore their significance to the method, have been described in relevant sections of the *Results*. The present study is part of the FREIA project (funded by EU Horizon 2020) which aims to safeguard female fertility by developing a multifaceted screening process, including *in-vivo*, *in-vitro*, and *in-silico* methods, to identify EDCs that cause female reproductive toxicity (van Duursen et al., 2020).

**TABLE 1:** Aspects of oocyte maturation and developmental competence assessed in the bovine oocyte model, described in this study.

Endpoint	Marker of
Nuclear maturation	Oocyte maturation
Mitochondrial redistribution	Cytoplasmic oocyte maturation
Cumulus cell expansion	Cumulus cell function and quality
Apoptosis	Cumulus cell function and quality
Steroid hormone production	Cumulus cell function and quality
Embryo production	Developmental competency

## 2 Materials and Equipment

### 2.1 Oocyte Recovery and Maturation

NaCl 0.9% (Braun, Germany).

Penicillin Streptomycin (GIBCO, Grand Island, New York, United States).

Medium M199 HEPES #22340 (GIBCO, Grand Island, New York, United States).

#### 2.1.1 Maturation Medium Composition

Medium M199 with Earle’s salts and glutamine #31100-027 (GIBCO, Grand Island, New York, United States) is buffered with 2.2% (g/L) NaHCO_3_, osmolarity is set at 280–290 mOsmol, and the medium is sterilized through a 0.22 um filter. The buffered medium is supplemented prior use with 10 IU/ml penicillin (GIBCO, Grand Island, NY, United States), 10 IU/ml streptomycin (GIBCO, Grand Island, NY, United States), 0.05 IU/ml rec hFSH (Organon, Jersey City, NJ, United States), 0.1 μM cysteamine M9768 (Sigma-Aldrich, St. Louis, MO, United States) and 10 ng/ml EGF E4127 (Sigma-Aldrich, St. Louis, MO, United States).

### 2.2 *In Vitro* Fertilization

Sperm was purchased from CRV, Arnhem, Netherlands. Breed of Meuse-Rhine-Yssel (MRY). All experiments were performed with sperm from the same bull with identification number NLDM000264706704.

Percoll^®^Plus GE 17-5445-02 (GE Healthcare, Chicago, IL, United States). The recipe for Percoll 90% and 45% solutions is listed in the Supplementary Material.

Rinsing medium R (Supplementary Material).

Sperm medium (Supplementary Material).

Fertilization medium (Supplementary Material).

Heparin H3393 (Sigma-Aldrich, St. Louis, MO, United States) (0.25 mg/ml in 0.9% NaCl).

Penicillamine-Hypotaurine-Epinephrine (PHE) (Supplementary Material).

### 2.3 *In Vitro* Embryo Culture

SOF medium composition (Supplementary Material).

### 2.4 Culture dishes and plates.

Thermo Scientific™ Nunc™ 4-Well Dishes for IVF (Thermo Fisher Scientific, Waltham, MA, United States).

CELLSTAR^®^ plate, 96 wells, F-bottom, #655180 (Greiner Bio-One, Germany).

Versaplate-Plexa 96 well-plate (Agilent Technologies, Santa Clara, CA, United States).

### 2.5 Equipment

Nikon Eclipse Ti-E inverted microscope with A1R confocal module (Nikon, Tokyo, Japan).

CentriVap Benchtop Vacuum Concentrator (Labconco, Kansas City, MO, United States).

AB Sciex 6500+ triple quadrupole mass spectrometer (Sciex, Framingham, MA, United States).

### 2.6 Other

Dimethyl Sulfoxide D2650-5X (Sigma-Aldrich, St. Louis, MO, United States).

Diethylstilbestrol (>99%, HPLC) D4628-1G (Sigma-Aldrich, St. Louis, MO, United States).

Paraformaldehyde 16% (Electron Microscopy Sciences, Hatfield, PA, United States).

4′,6-diamidino-2-phenylindole (DAPI) D9542 (Merck, Kenilworth, NJ, United States).

Vectashield (LSBio, Seattle, WA, United States).

Mitotracker Orange CMTMRos M7510 (Thermo Fischer Scientific, Waltham, MA, United States).

*In Situ* Cell Death Detection Kit, Fluorescein (Roche, Switzerland).

Ethidium Homodimer-1 (Invitrogen, Waltham, MA, United States).

Hoechst 33342 B2261 (Sigma-Aldrich, St. Louis, MO, United States).

## 3 Methods

The method we describe here aims to combine already established protocols in a coherent workflow that reflects pivotal processes in bovine oocyte maturation and embryo development. Carrying out this protocol should be possible in a well-equipped laboratory with access to confocal microscopy and to liquid chromatography - mass spectrometry (LC-MS). For the clarity of procedures, the protocol for obtaining mature cumulus-oocyte complexes (COCs) and blastocysts is described first, followed by each protocol targeting a specific endpoint using diethylstilbestrol (DES) as the test compound. Composition of all the media used is detailed in the *Materials and Equipment* section and the accompanying Supplementary Material.

### 3.1 *In Vitro* Embryo Production

The following protocol is the established protocol used routinely in the lab and described previously, with some modifications, by Brinkhof et al. (2015), and involves three phases:

#### 3.1.1 Oocyte Collection and *In Vitro* Maturation

Bovine ovaries obtained from the Gosschalk slaughterhouse (Epe, Netherlands) were transported in a thermos flask immediately after excision, washed with water and kept stably at 30°C in a 0.9% NaCl solution supplemented with 0.1% v/v penicillin-streptomycin. Cumulus-oocyte complexes (COCs) are aspirated, along with follicular fluid, from antral follicles (2–8 mm diameter) with a winged infusion set (18-gauge needle) connected to a 50 ml conical tube under vacuum. The sediment containing the COCs is transferred with a glass Pasteur pipette to a petri dish and examined under a stereomicroscope to isolate individual cumulus-oocyte complexes (COCs). COCs are selected based on morphological criteria; the presence of a healthy cumulus investment and a slightly darkened ooplasm. COCs with signs of late atresia, very dark ooplasm, or expanded cumulus with punctate dark spots, are excluded. The selected COCs are collected in clear follicular fluid and then washed twice in HEPES-buffered M199 media. This is followed by a third rinse with pre-incubated maturation medium. The COCs, in groups of 35–70, are transferred in 500 µl of pre-incubated maturation medium in a 4-well plate and placed in a 5% CO_2_ incubator at 39°C for 22–24 h. The minimum and maximum number of COCs (35 and 70, respectively) cultured in 500 μl media in a 4-well Nunc plate well were determined after optimization of the *in vitro* oocyte maturation protocol described here. When protocols require a smaller number of COCs than this minimum, the media volume is accordingly adjusted to maintain the ratio of media volume per COC (min-max). The use of a defined-composition medium with no steroid hormones present is essential when investigating the effects of endocrine disrupting chemicals.

#### 3.1.2 *In Vitro* Fertilization

After 22–24 h of IVM, frozen sperm, originating from the same bull (details in Methods) for all experiments and stored in a cryopreservation straw in liquid nitrogen after collection, is thawed for 1 min at 39°C. It is then aseptically deposited for separation in a Percoll^®^ gradient (45% and 90%) and centrifuged at x700G for 30 min at 27°C. In the meantime, the COCs are washed three times in pre-warmed rinsing media R. The Percoll^®^ gradient is aspirated carefully leaving the sperm pellet, which is resuspended in 50 µl of pre-warmed sperm medium. The rinsed COCs are then placed in 430 μl of pre-incubated fertilization medium per well in a 4-well plate and then supplemented with heparin (final concentration 10 μg/ml) and PHE (detailed in Supplementary Material). Sperm is added at a final concentration of 1 × 10^6^/ml (optimized per bull) to co-incubate with the COCs for 18–22 h in a 5% CO_2_ incubator at 39°C.

#### 3.1.3 *In Vitro* Embryo Culture

The next day after IVF, day 1 of IVC, 500 µl synthetic oviductal fluid (SOF) medium is placed per well in a 4-well plate and pre-warmed in a 5% CO_2_, 7% O_2_ incubator. The presumably fertilized oocytes are denuded from the cumulus cell layer by vortexing in 1 ml of pre-warmed rinsing medium R for 3 min. The denuded oocytes/zygotes are collected, washed twice in pre-warmed rinsing medium R, transferred to the SOF medium (supplemented with the EDC when assessing effects on embryo development), and placed in a 5% CO_2_, 7% O_2_ incubator. At day 5 of IVC, the embryos are categorized based on cell number as uncleaved, ≤8 cell embryos and, >8 cell embryos. The uncleaved ones are discarded and the rest are transferred to new pre-incubated SOF medium (supplemented with the EDC when assessing effects on embryo development). Blastocyst numbers are recorded at Day 7 and Day 8 post fertilization.

### 3.2 DES Exposure and Endpoints

In total, six different endpoints are investigated here to assess the effect of DES on the maturation of oocytes and the production of embryos in the bovine model. A stock solution of the chemical is made with the appropriate dissolvent (vehicle), stored appropriately, and renewed regularly. When making working solutions complete solubility of the chemical in the maturation medium should be ensured. Maturation medium or SOF medium, here, was supplemented with DES dissolved in dimethyl sulfoxide (DMSO) at final concentrations of 10^–9^ M, 10^–7^ M, and 10^–5^ M. For assessing effects of DES on oocyte developmental competence, the timing of exposure is restricted to the 24 h of IVM. For assessing effects on early embryo development, exposure to DES was performed during the 8 days of IVC. For all experiments a control group with no DES or DMSO added, and a blank group containing the vehicle DMSO (0.01% v/v), were included.

#### 3.2.1 Detection of DNA Organization After DES Exposure During Oocyte Maturation

**FIGURE 1:**
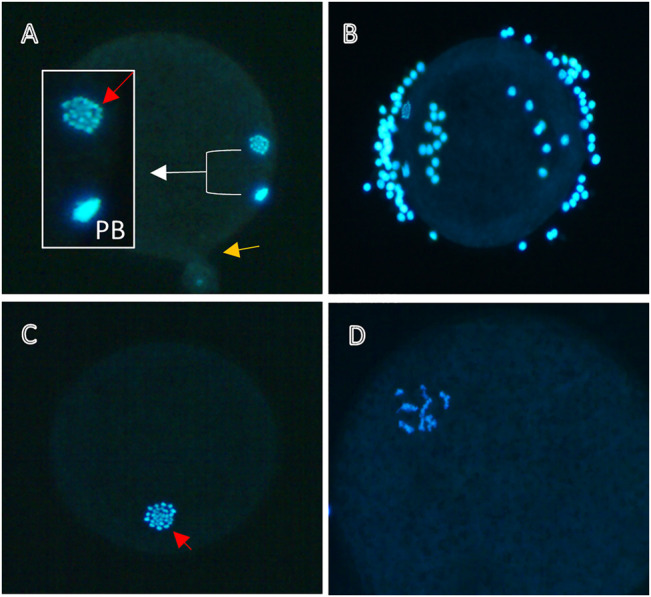
Nuclear maturation of bovine oocytes. Representative images of denuded oocytes matured *in vitro* for 24 h without **(A,B)** or with **(C,D)** 10^−5^ M DES and stained with DAPI for DNA visualization. **(A)** Mature oocyte with a clear metaphase plate (red arrow) and a polar body (PB). The yellow arrow denotes the point of rupture, **(B)** Insufficient denudation may obstruct the view of the nucleus (visible here), thus not allowing for a classification of the meiotic status. Oocytes treated with EDCs like DES might arrest in earlier cell cycle stages, for example in MI **(C)**, or exhibit abnormal chromatin patterns **(D)**.

Nuclear maturation can be detected by the presence of a metaphase plate (MII) and the extrusion of the first polar body. COCs obtained after IVM are denuded of their cumulus cells (as described in Section 3.1.3) and fixed for 15 min in 4% paraformaldehyde (PFA). The denuded oocytes are then stained with DAPI (0.1 μg/ml) for 1 min and mounted in Vectashield mounting medium on a slide with petroleum jelly bridges. The slides are then sealed and examined under a fluorescence microscope. The oocytes are characterized as MII when they have a clear metaphase plate and a polar body (or condensed DNA destined to be extruded as the polar body) or as abnormal when they are arrested at the GV-stage, in other earlier than MII meiotic stages, or have a degenerated chromatin configuration (for examples see Figure 1).

#### 3.2.2 Mitochondrial Distribution After DES Exposure During Oocyte Maturation

Mitochondrial staining of oocytes is performed with the membrane-potential dependent stain Mitotracker™ Orange CMTMRos (#M7510). Denuded oocytes are incubated in maturation medium supplemented with 200 nM Mitotracker™ Orange CMTMRos for 30 min at 39°C in a 5% CO_2_ incubator. After washing in PBS (0.1% w/v PVP), the oocytes are fixed in 2% PFA in PBS for 15 min, washed again and stained with the DNA dye DAPI (0.1 μg/ml) for 3 min. The oocytes are mounted on glass slides in Vectashield and samples are stored at 4°C in the dark until imaging by confocal microscopy at a Nikon A1R confocal microscope. A 488 nm laser and 490–520 nm emission range are used for chromatin visualization by DAPI staining, and the 561 nm laser and 554–645 nm emission filter for mitochondria visualization. Z-stack images are obtained with a step-size of 2 µm. The distribution of Mitotracker red fluorescence in the center of the oocyte (the inner 80% of the cell) versus the cortex region of the oocyte (the 10% periphery on both sides of the cell) was quantified using Image J software.

#### 3.2.3 DES Exposure and COC Expansion During Oocyte Maturation

For COC expansion quantification, IVM is carried out as described in Section 3.1 with small modifications. COCs are matured as groups of 7–10 per well in 100 µl of maturation medium in a 96-well flat-bottom plate. The number of COCs needs to be reduced to avoid clumping, so images (x50) of individual COCs can be acquired in a camera-fitted stereomicroscope, at the start and end of IVM. The quantification of the expansion was carried out as previously described in Solak et al. (2014). The images are analyzed with the Adobe Photoshop CC2018 software. Each COC is selected with the Object selection tool that demarks the outline of COCs, and the area size is calculated in pixels. The ratio of the projected surface area of each COC after and before IVM represents the fold-increase of the COC.

#### 3.2.4 Apoptosis Detection and Quantification After DES Exposure During Oocyte Maturation

Exposure of an oocyte to a chemical might trigger a stress-response and eventually lead to apoptotic events. The toxic potential of an EDC on a COC can be ascertained by the use of an apoptosis detection kit. The *In Situ* Cell Death Detection kit, based on the Terminal deoxynucleotidyl transferase dUTP nick end labeling (TUNEL) assay, can be used with slight modifications to allow for use on oocytes for the detection of DNA fragmentation. All washing steps included are performed in 0.3% PVP/PBS for 5 minutes. A positive control of COCs treated for 15 min with DNase, and a negative control of COCs incubated only with the labeling solution, should always be included.

In a 96-well plate COCs are washed three times and then incubated for 5 min in 4 μM Ethidium homodimer-1 (EthD-1) diluted in PBS. The COCs are fixed in 4% PFA for 15 min, transferred to the permeabilization solution (0.1% v/v Triton X-100/0.1% w/v sodium citrate/PBS) and incubated on ice for 5 min. The COCs are placed in the TUNEL reaction mixture for 1 h at 37°C in the dark, as per the kit manufacturer instructions. DNA is stained with Hoechst (10 μg/ml) for 30 min and the COCs are mounted in Vectashield. Between all aforementioned steps the COCs were washed at least three times. The analysis of the samples is performed on a confocal fluorescence microscope using three separate channels for EthD-1 (exc. 561 nm with emission at 580–650 nm), TUNEL (exc. 488 nm with emission 500–550 nm), and Hoechst (exc. 405 with emission at 420–490 nm). An optical z-stack with 10 um steps is acquired and three separate optical planes of each COC are selected to process. Hoechst-, TUNEL-, and EthD-1-positive nuclei are counted with ImageJ after background subtraction and watershed separation with the analyze particles function based on adjusted fluorescence threshold and particle size. A ratio of TUNEL-, and EthD1-positive nuclei to the total cell number (Hoechst) provides the rate of apoptosis and necrosis, respectively. Nuclei that are positive for both EthD1 and TUNEL are considered as secondary apoptotic/necrotic. Particular caution should be taken when examining nuclei with “dots” of TUNEL staining overlapping with DAPI as these might suggest initial stages of DNA fragmentation.

#### 3.2.5 Quantification of Steroid Hormones Secreted in Maturation Media During DES Exposure

Oocytes in groups of 70 are matured in 500 μl of maturation media as described previously, with or without DES, and the conditioned media is collected at the end of IVM, snap frozen in liquid nitrogen and thawed to room temperature prior to solid phase extraction (SPE). 100 µl of the culture medium was weighed and used for the analysis with the addition of 50 μl of corresponding steroid internal standard. The SPE extraction was done using Versaplate-Plexa 96 well-plate with 30 mg cartridges. The cartridges were pre-conditioned, subsequently the samples were loaded and then the cartridges were washed with 30% methanol. Finally, the steroids were eluted with methanol. The extracts were evaporated to dryness at 40°C using the CentriVap Concentrator and reconstituted to 50% methanol. The LC-MS/MS analysis was conducted using AB Sciex 6500+ triple quadrupole mass spectrometer that was coupled to the corresponding AB Sciex Exon UPLC system. Positive electrospray ionization (ESI) was applied. The chromatographic separation was achieved on a Phenomenex Kinetex C18 reverse phase column (150 × 3 mm, 2.6 μm particle size, pore-size 100 Å) with column oven at 40°C. Mobile phase A consisted of Milli-Q water with the addition of 0.2 mM ammonium fluoride solution and mobile phase B consisted of methanol. The sample injection volume was 10 μl. Quantification of steroid hormones was done by isotope-dilution mass spectrometry using multi-reaction monitoring (MRM).

An additional derivatization reaction with dansyl chloride was included for estrogen detection. Dansyl chloride diluted in acetone was combined with bicarbonate buffer (pH 10.5) in a quantity 1:1, the plate was shaken well and the reaction was conducted at 60°C for 4 min. Then the samples were analyzed again with the same settings as mentioned above.

Validation of the SPE method showed the following recoveries range for each steroid at the level of 3 ng/ml: 74%–78% for pregnenolone, 112%–124% for progesterone, 136%–154% for 17-hydroxyprogesterone, 111%–115% for testosterone, 131%–149% for 5α-androstenediol, 116%–120% for androstenedione, 115%–124% for corticosterone, 100%–107% for 11-deoxycorticosterone, 156%–174% for 11-deoxycortisol, 117%–124% for cortisol, 118%–122% for cortisone. In addition, at the level of 25 pg/ml recoveries for the dansylated estrogens were 95%–107% for dansylated estrone and 89%–98% for dansylated 17β-estradiol.

The SPE LC-MS/MS method has been corroborated using an established radioimmunoassay (RIA) for quantifying progesterone and estrogen levels (Aardema et al., 2013a). These data as well as the method are included in Supplementary Figure S1.

#### 3.2.6 Developmental Competence and Embryo Development Assessment After DES Exposure During Oocyte Maturation or Embryo Culture

During *in vitro* maturation, the oocyte acquires developmental competence. By carrying out IVM in the presence of DES the effect on developmental competence may be examined after IVF and IVC of the DES-exposed oocytes. Cleavage rates are determined on day 5, and blastocyst numbers on Day 7 and Day 8 of IVC (for protocol see Section 3.1.3). Developmental competence can be assessed by the blastocyst rate measured as the number of blastocysts produced per fertilized oocytes. Conversely, to assess effects of DES on early embryo development, developmental rates of embryos, produced from fertilized oocytes that were not exposed to DES during IVM, can be monitored while being exposed to DES during the 8 days of IVC.

### 3.3 Statistical Analysis

Data were analyzed with the IBM SPSS vs25 statistics software to compare the groups with one-way ANOVA followed by a Tukey’s post hoc test. Data on nuclear maturation were analyzed with the chi-square test followed with post hoc Bonferroni corrected *z*-tests. Data on apoptotic and necrotic rates were analyzed with the Kruskal–Wallis test followed by pairwise comparisons performed using Dunn’s (1964) procedure. At least three biological replicates are performed for each experiment. In the figures the total number of oocytes or fertilized oocytes analyzed per experimental group are provided. Specific number of oocytes or fertilized oocytes analyzed per independent experiment are included in Supplementary Table S1. Comparisons reported are between DES-exposed groups with the vehicle-treated group. Statistical significance is defined as *p* < 0.05.

## 4 Results

### 4.1 Effect of DES Exposure on Nuclear Maturation

Mammalian oocytes are enclosed in follicles within the ovary, and are arrested at the prophase stage of meiosis I. At each menstrual cycle, some of the follicles are triggered to enter the pool of growing follicles. In mono-ovulatory species, a single Graafian, or dominant, follicle will be able to ovulate and release a mature cumulus-oocyte complex (COC) (Rüsse, 1983; Fair, 2003). In mammals, like the cow, the oocyte in this COC has completed the first meiotic division (with a characteristic first polar body extrusion) and the homologous chromosomes are aligned in the equatorial plane of the metaphase spindle of the second meiotic division. This so-called nuclear maturation of oocytes is triggered by the midcycle surge of luteinizing hormone (LH) and the first event denoting it is the breakdown of the germinal vesicle (GVBD). The basic molecular mechanisms controlling meiotic arrest at the prophase stage and timely resumption of meiosis are reviewed in Pan and Li (2019). There is evidence that nuclear maturation may be partially regulated by gonadotropin-dependent estradiol signaling. Specifically, estrogen was shown to regulate the natriuretic peptide receptor 2 (NPR2) of the natriuretic peptide C (NPPC)/NPR2 system, that is responsible for the maintenance of meiotic arrest (M. Zhang et al., 2011; Jaffe and Egbert, 2017; Liu et al., 2017; Xi et al., 2018). This implies that oocyte nuclear maturation is susceptible to EDC effects with estrogenic action. DES specifically has been previously shown to inhibit meiosis in *in vitro* cultured mouse oocytes, although possibly by inducing ER-independent abnormalities in spindle assembly (Ding et al., 2020).

#### 4.1.1 Principle of Method

Typically, around 80%–90% of bovine oocytes matured *in vitro*, as described in Section 3.1, are expected to reach metaphase II (MII). EDCs like DES may inhibit meiotic progression by, for example, interfering with spindle assembly. Figure 1 depicts two bovine oocytes that were DAPI stained after maturation *in vitro* in absence of DES exposure (Figures 1A,B), and two that were DAPI stained after exposure to 10^–5^ M DES (Figures 1C,D). The emergence of a clear metaphase plate (MII) and a first polar body (PB) are the two characteristics of what is defined here as a mature oocyte (Figure 1A). Oocytes not achieving nuclear maturation (abnormal) were identified when they were at stages of meiosis earlier than MII (e.g., MI; Figure 1C) or when chromatin conformation was abnormal (Figure 1D).

#### 4.1.2 Results

Bovine oocytes were exposed to DES for 24 h during IVM and, after DAPI staining, classified as MII or abnormal. The low and middle concentrations of DES (10^–9^ M, 10^–7^ M) had no negative impact on the oocytes’ ability to resume meiosis up to the MII stage (Table 2). In contrast, exposure to a concentration of 10^–5^ M DES almost completely blocked oocyte maturation (3% at MII, *n* = 97). The majority of these oocytes had abnormal chromatin patterns, seemingly dispersed chromosomes in the cytoplasm, similar to the one shown in Figure 1D. In some cases, the cell cycle was arrested at the MI-stage, or DNA was highly condensed.

**TABLE 2:** Effect of diethylstilbestrol (DES) on the nuclear maturation of *in vitro* matured bovine oocytes.

	Experimental group						
			Control	0.01% DMSO	10^–9^ M	10^–7^ M	10^–5^ M
Meiotic Status	MII	Count*	72	76	72	81	3
		%	80^a^	84^a^	82^a^	80^a^	3^b^
	Abnormal	Count*	18	14	16	20	94
		%	20^a^	16^a^	18^a^	20^a^	97^b^

MII, metaphase plate.

*Total count of three independent experiments (Supplementary Table S1).

a,b Values in the same row without a common superscript letter are statistically significantly different (*p* < 0.05).

#### 4.1.3 Technical Aspects and Limitations

Technically this protocol does not present challenges for a trained researcher, but there are two important aspects to take into consideration. First, to avoid loss of chromatin from the oocyte (MII or PB), pressure on the coverslip after oocyte mounting on a slide should be modulated to prevent rupture which allows the ooplasm to seep through the oocyte membrane (Figure 1A, yellow arrow). This can be counteracted by the use of an adhesive spacer between the slide and the coverslip. Second, complete denudation of the oocyte is important to ensure that the view of the nucleus or of the PB is not obstructed by cumulus cells (Figure 1B). The use of the enzyme hyaluronidase may be opted to facilitate denudation. The simplicity of the process and the materials in use have made this protocol a widely used tool to evaluate nuclear maturation of oocytes by many researchers. Nevertheless, the experience needed for the identification of meiotic stages, should be taken into account to ensure accuracy and reproducibility of data. To gain more insight into mechanistic aspects, nuclear stages of all oocytes (e.g., anaphase, pro-metaphase et cetera) might be recorded to identify specific meiotic blocks.

### 4.2 Effects of DES Exposure on Mitochondrial Distribution

Concurrent with nuclear maturation are changes within the oocyte cytoplasm, generally termed as cytoplasmic maturation. Both nuclear and cytoplasmic maturity are required for an oocyte to become developmentally competent to produce an embryo upon fertilization. Cytoplasmic changes include accumulation of maternal transcripts, re-organization and relocation of cytoplasmic organelles (e.g., Golgi network, endoplasmic reticulum, etc.). For example, in the bovine oocyte, mitochondria redistribute from a peripheral position to a more diffused pattern upon MII-arrest (Ferreira et al., 2009). Potentially, EDC action interferes with cytoplasmic aspects of oocyte maturation as well. Indeed, EDCs such as phthalates and polychlorinated biphenyls (PCBs) have been described to perturb the redistribution and function of cytoplasmic organelles of *in vitro* matured bovine oocytes and *in vivo* after oral administration in mice (Pocar et al., 2001; Kalo and Roth, 2015; Li et al., 2019; Lu et al., 2019). The effects of DES on cytoplasmic maturation remain to be elucidated.

#### 4.2.1 Principle of Method

**FIGURE 2:**
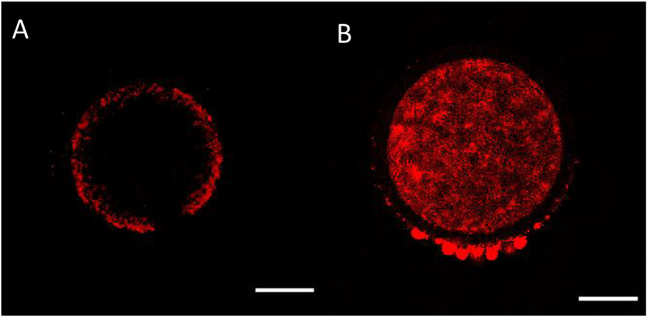
Mitochondrial distribution in bovine oocytes visualised by Mitotracker Orange CMTMRos staining. **(A)** Peripheral mitochondrial distribution in an immature oocyte. **(B)** Diffused mitochondrial distribution in a mature oocyte. The scale bar represents 50 μm.

**FIGURE 3:**
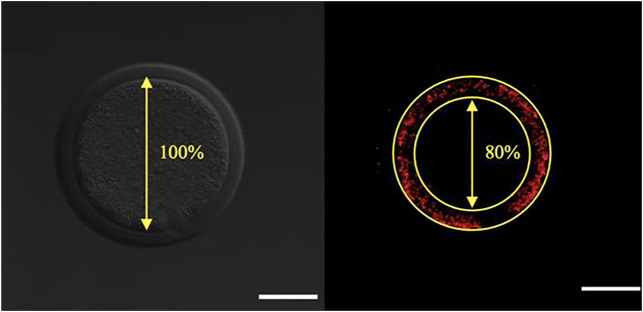
Calculation of the percentile center size of an oocyte based on mitochondrial distribution visualized by Mitotracker Orange CMTMRos staining before final oocyte maturation. Left panel: Denuded oocyte, bright fluorescence field image corresponding to the oocyte on the right. Right panel: The designated center, denoted by the encircled area with absence of fluorescence, is recorded and expressed as a percentage of the total oocyte diameter. The scale bar represents 50 μm.

One of the aspects of cytoplasmic maturation in the bovine oocyte is the redistribution of mitochondria from a peripheral position at the germinal vesicle stage (Figure 2A) to a centrally diffused position during oocyte maturation (Figure 2B). The peripheral distribution of mitochondria in immature oocytes forms a clear ring-like shape that can be used to calculate the percent diameter that it occupies within the oocyte (Figure 3). The equator of the immature oocyte is found at the maximum optical X-Y projection of the optical Z-section of the oocyte. For example, in Figure 3 (right panel), the mitochondria of this immature oocyte occupy 20% of the oocyte’s diameter (10% in each side), which is considered as the periphery or cortex, and the rest (80% of total diameter) is considered to be the center. The redistribution of mitochondria in mature oocytes can be quantified as the ratio of fluorescence intensity per pixel of the periphery to the fluorescence intensity per pixel of the center (previously spatially defined as percentage of diameter in immature oocytes). The mean fluorescence intensity per pixel (arbitrary units) in each spatial compartment is calculated with ImageJ. The ratio of peripheral versus central fluorescence intensity is expressed in relative peripheral intensity compared to the center to assess the distribution pattern of mitochondria.

#### 4.2.2 Results

**FIGURE 4:**
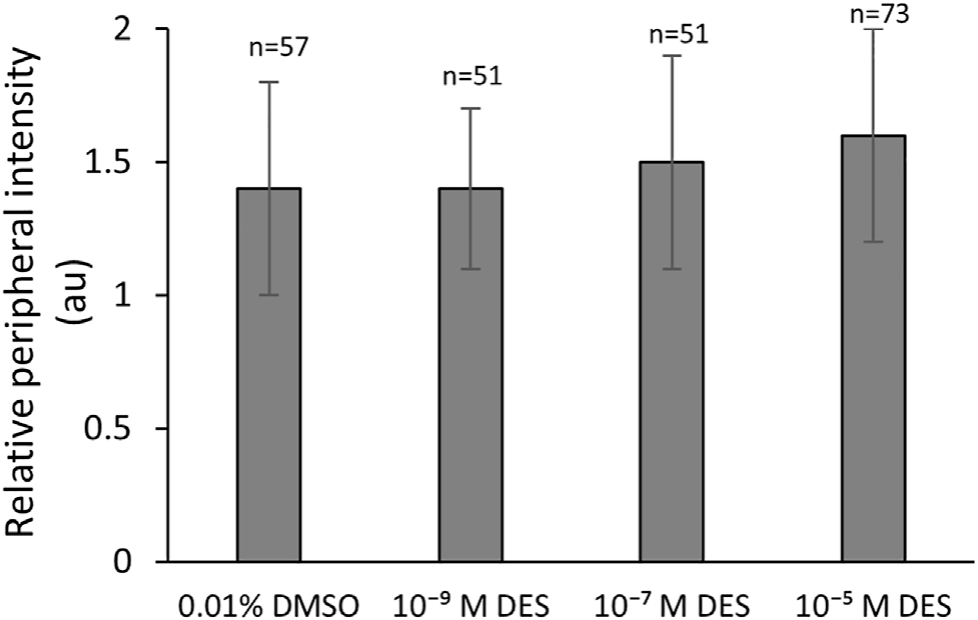
Mitochondrial redistribution during *in vitro* bovine oocyte maturation is not affected by DES. Bars represent mean of three replicates (±SD). Numbers (n) indicate total count of three independent experiments.

Bovine oocytes matured *in vitro*, with or without DES, were stained with the membrane-potential-dependent stain Mitotracker Orange CMTMRos. The ratio of the fluorescence intensity per pixel of the peripheral area to the fluorescence intensity per pixel of the central area, represents the active mitochondria distribution pattern (peripheral or cortex vs. central). In mature oocytes, there were no significant effects of DES exposure during IVM on the distribution of active mitochondria (cortex vs. center) at any of the concentrations tested. The relative peripheral intensity was similar between non-exposed (0.01% DMSO, 1.4 ± 0.4 Arbitrary Units - AU) and DES-treated groups (1.4 ± 0.3, 1.5 ± 0.4, 1.6 ± 0.4 AU, ascending concentrations) (Figure 4).

#### 4.2.3 Technical Aspects and Limitations

The described method makes use of the membrane-potential dependent dye Mitotracker Orange CMTMRos, thus fluorescence intensity per pixel measured for each oocyte is dependent on the aerobic activity of mitochondria. Consequently, data from this dye do not consist a representation of the total mitochondrial complement but rather a representation of aerobically active mitochondria. Interpretation of the data should be made accordingly or another dye that is not membrane-potential dependent might be used instead.

### 4.3 Effects of DES Exposure on COC Expansion

In the adult intraovarian environment, the maturing oocyte is surrounded by somatic components of the follicle, namely the theca, granulosa, and cumulus cells (CC) that support the maturation process. The cumulus layer and the enclosed oocyte share a dynamic bidirectional relationship through paracrine factors and directly through transzonal projections (TZPs) (Albertini et al., 2001; Hussein et al., 2005; Macaulay et al., 2016). During the final stages of oocyte maturation, the CC layer expands through the secretion of a hyaluronic acid (HA)-rich extracellular matrix in response to epidermal growth factor (EGF) ligands produced by LH-stimulated granulosa cells (Park et al., 2004). In mice, inhibition of HA synthesis leads to failure of CC expansion associated with anovulation, suggesting an important role in ovulation (Chen et al., 1993). An exact mechanism of the role of CC expansion in ovulation has not been elucidated yet, but it has been observed in mice that CCs upon expansion adopt a migratory and invasive phenotype that potentially, *in vivo*, might contribute to follicular wall rupture (Akison et al., 2012). The degree of CC expansion has also been linked to the developmental potential of bovine oocytes (Aardema et al., 2013b). CC expansion may be affected by the action of EDCs, as supported by the example of bisphenol A (BPA) that was reported to reduce the degree of CC expansion of mouse, bovine, and porcine oocytes exposed during *in vitro* culture (Mlynarcíková et al., 2009; Acuña-Hernández et al., 2018; Saleh et al., 2021).

#### 4.3.1 Principle of Method

**FIGURE 5:**
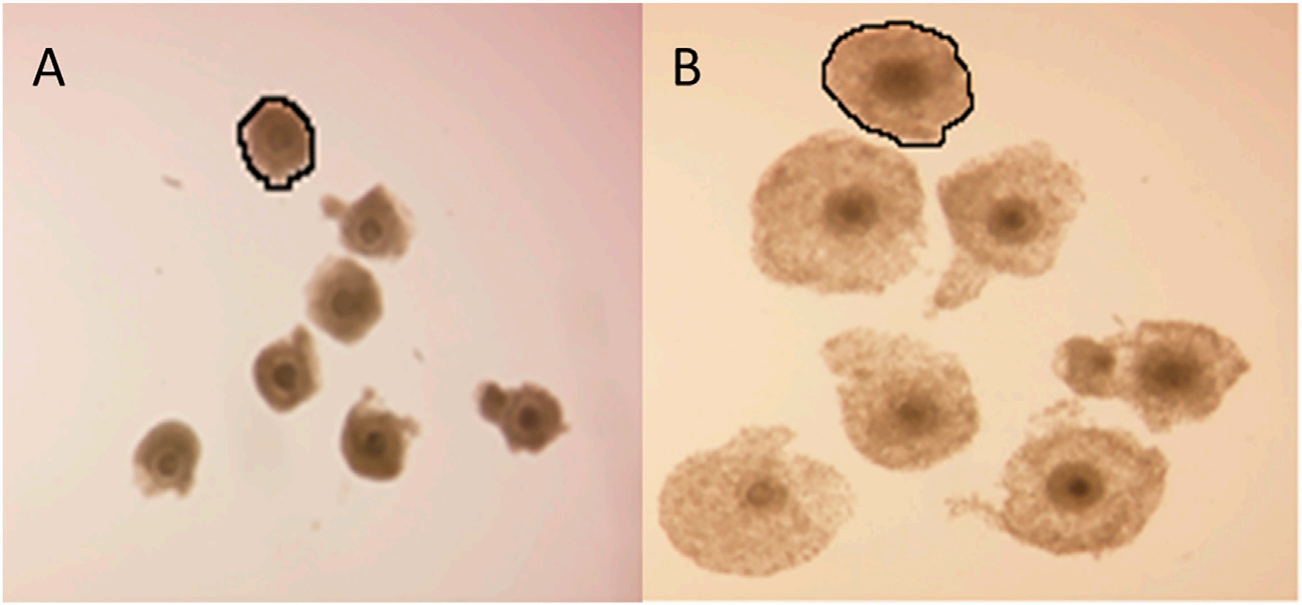
Cumulus cell expansion. Representative photomicrographs of bovine COCs before and after IVM. Fold-increase of the encircled COC can be calculated as the ratio of the projected surface area before **(A)** and after **(B)** IVM in pixels.

Cumulus cell expansion occurs during the final stages of oocyte maturation through the synthesis of a hyaluronic acid-rich extracellular matrix (ECM). The measurement of cumulus cell expansion, either quantitatively or in defined descriptive categories, has been widely adopted as a COC quality indicator. COC expansion was determined before and after the IVM procedure and the fold-increase of the COCs as the ratio of projected surface area of a COC before and after IVM was calculated from the respective amount of pixels. As an example, the encircled COC of Figure 5A had a projected surface area of 13,231 pixels and this same COC after 24 h of IVM (Figure 5B) had a surface area of 38,061 pixels, thus the COC expanded 2.8-fold.

#### 4.3.2 Results

**FIGURE 6:**
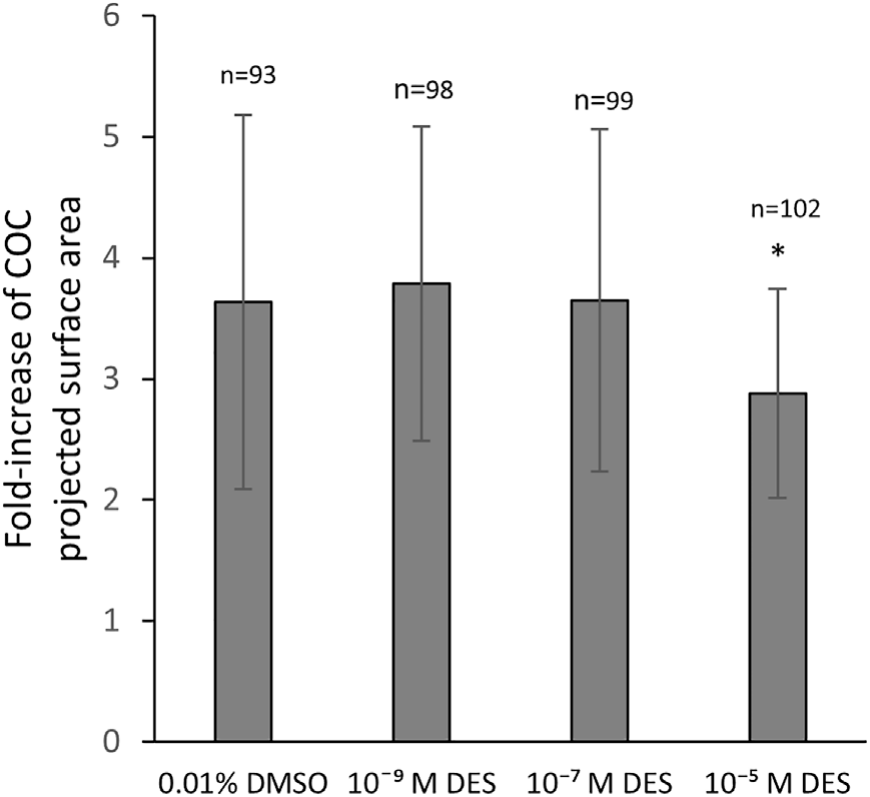
Cumulus layer expansion upon DES exposure. Exposure of COCs to high concentrations of DES reduces the magnitude of cumulus expansion (*p* < 0.05). Low and middle DES concentrations had no effect in COC expansion. Bars represent mean of three replicates (±SD). *Statistically significantly different from vehicle-treated COCs (*p* < 0.05). Numbers (n) represent total count of three independent experiments.

2Exposure of COCs to DES during IVM statistically significantly impacted the degree of COC expansion (Figure 6). The vehicle-treated group (0.01% DMSO) showed a mean 3.6-fold increase of the projected COC surface area. COCs that were exposed to low (10^–9^ M) and middle (10^–7^ M) concentrations of DES had a similar mean fold-increase of 3.8 ± 1.4 and 3.6 ± 1.4, respectively (Figure 6). COCs exposed to the highest DES concentration of 10^–5^ M, had a statistically significantly (*p* < 0.05) smaller expansion of 2.8 ± 0.9 -fold, compared to vehicle-treated controls (Figure 6).

#### 4.3.3 Technical Aspects and Limitations

The method followed here does not acount for the third dimension of a COC and does not represent the changes in volume of the asymmetrically shaped bovine COC. In addition, changes in oocyte volume versus cumulus expansion cannot be distinguished. Nevoral et al. (2015) have reviewed methods of cumulus cell expansion quantification, including measurement of the degradation products of hyaluronic acid. Despite the aforementioned limitations in tracking cumulus cell volume changes, the lack of refinement of other methods and the absence of preparatory steps in this non-invasive method described here, make it a good tool to evaluate cumulus cell expansion.

### 4.4 Effects of DES Exposure on Apoptosis

#### 4.4.1 Principle of Method

**FIGURE 7:**
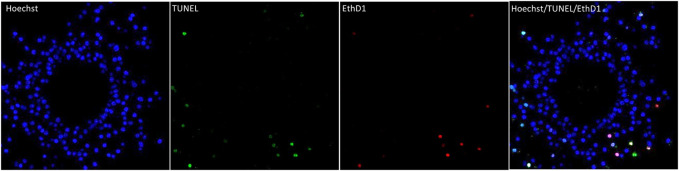
Apoptosis and necrosis detection in a bovine COC with the TUNEL assay (apoptosis) and EthD-1 stain (necrosis). The first panel shows the nuclei (blue, Hoechst, all nuclei) of the cumulus cells in an optical z-section of a COC matured in vitro for 24 h. The chromatin of the oocyte (non-fluorescent circle in the middle) is not visible in this plane. The two middle panels show cells with fragmented DNA (green, TUNEL) and the necrotic (red, EthD1) cells and of the same optical section. The last panel is an overlay of the Hoechst, EthD1, and TUNEL channels. All panels show the same optical section of the same COC.

To evaluate possible effects of DES exposure on maturing COCs the apoptotic, and/or necrotic status of both cumulus cells and oocytes were evaluated. Mature COCs were stained with TUNEL and Ethidium homodimer-1 (EthD1), and their respective apoptotic and necrotic rates (% of apoptotic or necrotic nuclei per total nuclei as calculated from Hoechst staining) were quantified by confocal fluorescence imaging processing (Section 3.2.4). Figure 7, shows as an example a single cross-section of a vehicle-treated (0.01% DMSO) mature COC with 227 Hoechst stained cumulus cell nuclei, 10 necrotic nuclei (EthD1-positive ± TUNEL-positive) and seven apoptotic nuclei (only TUNEL-positive, out of 17 total TUNEL-positive).

#### 4.4.2 Results

**FIGURE 8:**
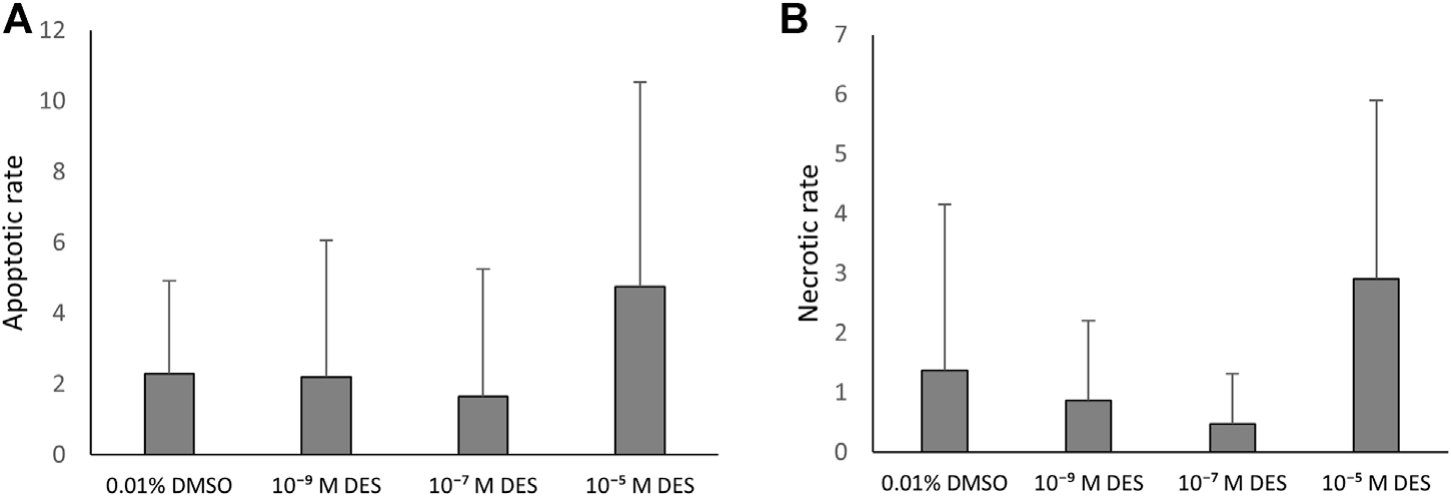
Apoptosis in the bovine COC is not affected by DES exposure. **(A)** The apoptotic rate is calculated as the number of TUNEL-positive/EthD1-negative cells per total cumulus cells number (Hoechst). DES exposure did not influence cumulus cell apoptotic rate. **(B)** The necrotic rate is calculated as the number of EthD1-positive ±TUNEL-positive cells per total cumulus cells number (Hoechst). The distribution of necrosis rate observed for the high DES group (10^−5^ M DES) was different from the vehicle-treated group (0.01% DMSO) (*p* < 0.001). Bars represent mean of three replicates (±SD). COCs and corresponding cumulus cells analyzed per group and per independent experiment are reported in Supplementary Table S1.

*In vitro* matured COCs exposed to DES were stained as described and due to the detection of multiple outliers within the dataset the non-parametric Kruskal–Wallis test was performed for statistical analysis. Apoptosis incidence was comparable between oocytes exposed to DES and oocytes matured in medium containing 0.01% v/v DMSO (Figure 8A). The necrotic rate analysis revealed a statistically significant difference in distribution of necrosis rates among the groups (*H*_(3)_ = 20.793, *p* < 0.001), and specifically between the oocytes of the vehicle-treated group and the 10^–5^ M DES-exposed oocytes (Figure 8B). Only the necrotic rate of cumulus cells was altered by DES exposure of maturing COCs, but levels of necrosis were still relatively low. The oocytes appear to be relatively resistant for apoptosis and necrosis since no TUNEL-positive or EthD1-positive oocytes were observed in any of the experimental groups.

#### 4.4.3 Technical Aspects and Limitations

The interpretation of the results ought to reflect that DNA fragmentation, detected by TUNEL assay, is a late event occurring during apoptosis and does not provide insights on the initiating event. In the absence of DNA fragmentation, it may be advisable to use other methods to detect earlier stages in the signaling cascade of apoptosis such as Western blot detection of activated caspases or the detection of phosphatidylserine in the outer leaflet of the plasma membrane (e.g., by annexin V staining).

### 4.5 Effects of DES Exposure on Steroidogenesis

An important function cumulus cells share, in a lesser extent, with the other follicle somatic cells is the production of steroid hormones that are implicated with meiotic progression and ovulation. Ovarian steroidogenesis relies on the theca and granulosa cells on which the gonadotrophins LH and FSH act, respectively. Androgens are produced from cholesterol in theca cells, and then converted to estrogens in granulosa cells. After ovulation, the granulosa cells undergo luteinization and a shift from estradiol to progesterone production occurs (Jamnongjit and Hammes, 2006). Some EDCs have the capacity to alter steroid hormone synthesis by inhibiting enzymes of the steroidogenic pathway, binding of hormones receptors, and by altering hormone secretion (Nam et al., 2003; Bloom et al., 2016; Hannon and Flaws, 2015). Abnormalities in ovarian steroid hormone production, synthesis, and systemic circulation is implicated in menstrual cycle irregularities and anovulation incidence (Kawa et al., 2021).

#### 4.5.1 Principle of Method

**FIGURE 9:**
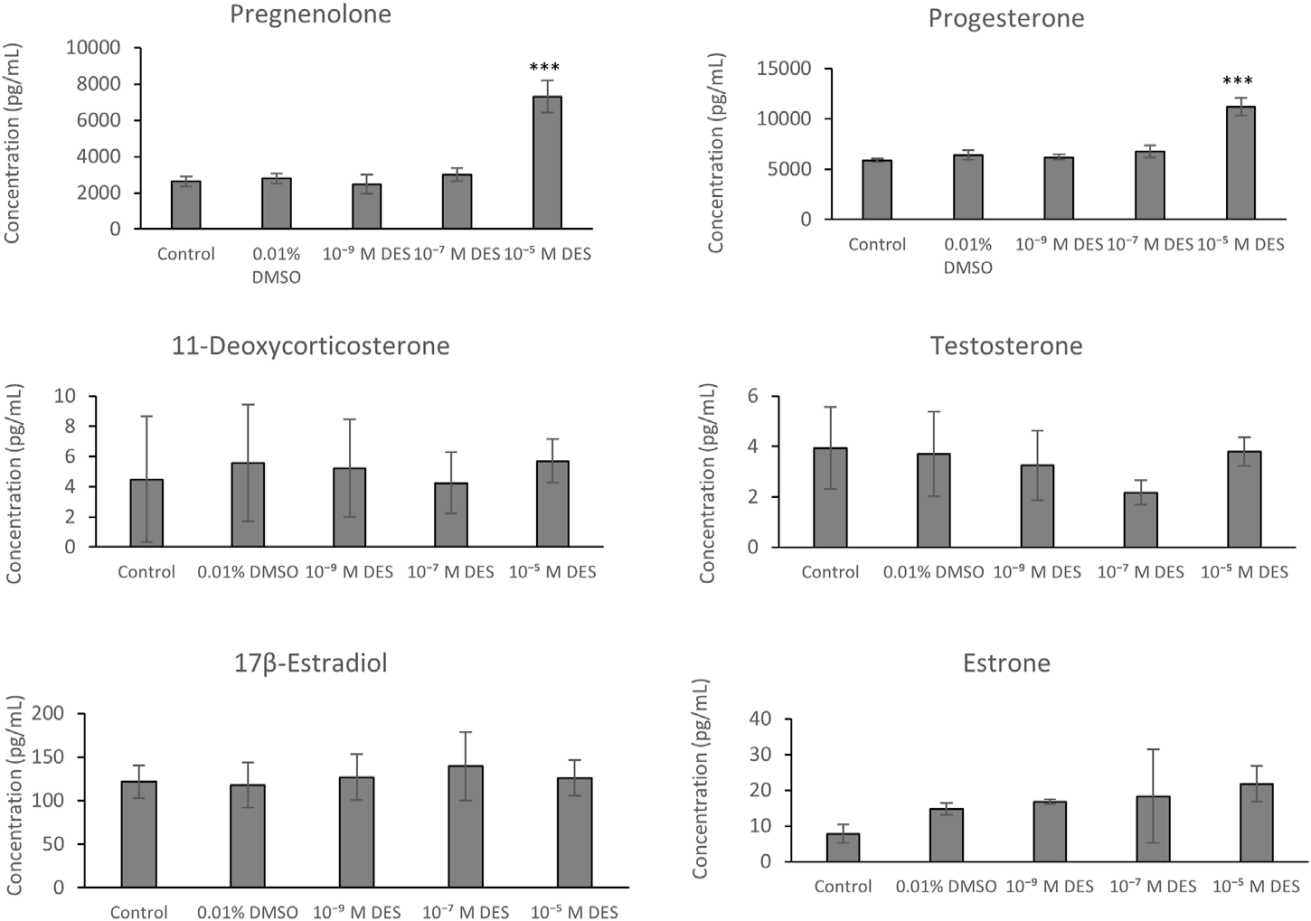
Steroid hormone levels in media collected from bovine COCs cultures exposed to DES or vehicle control (0.01% DMSO) or media without vehicle (control). Bars represent mean of three replicates (±SD). ***Statistically significantly different from vehicle-treated control (*p* < 0.001).

Cumulus cells of bovine oocytes are able to secrete steroid hormones in the maturation medium during IVM. The chemically-defined maturation medium, in which COCs were matured, was collected for each experimental group and steroid hormones were quantified by LC-MS/MS (Figure 9). The method included an analysis of 11-deoxycortisol, cortisol, cortisone, 17α-hydroxyprogesterone, androstenedione, 11-deoxycorticosterone, pregnenolone, progesterone, testosterone, estrone, and 17β-estradiol. Only the latter six steroid hormones were present in levels above the limit of detection.

#### 4.5.2 Results

Steroid hormone analysis revealed no statistically significant differences in levels of 11-deoxycorticosterone, testosterone, estrone, and 17β-estradiol between media samples obtained from DES-exposed COCs versus vehicle-treated COCs. There were also no statistically significant change in hormone synthesis between COCs cultured in media containing DMSO (vehicle) and media without vehicle (control). Pregnenolone was detected in higher concentrations in the media collected from COCs exposed to 10^–5^ M DES (7.30 ± 0.89 ng/ml) compared to media from the vehicle-treated group (2.79 ± 0.28 ng/ml) (Figure 9). Similarly, progesterone concentration was higher in the media collected from the high-DES group (11.20 ± 0.86 ng/ml vs. control 6.40 ± 0.47 ng/ml) (Figure 9). Additionally, potential changes in 17β-estradiol and progesterone levels were corroborated by a radioimmunoassay (Supplementary Figure S1).

#### 4.5.3 Technical Aspects and Limitations

LC/MS analysis was able to provide data on the levels of a wide variety of steroid hormones in the maturation media. Some steroid hormones were not present in levels above the limit of detection. For estrogens, sensitivity of the detection was increased, and LODs were decreased, by derivatization with dansyl chloride. One other important technical aspect to be followed is to ensure and facilitate the separation of steroids with similar masses and retention time, especially when assessing a broad steroidogenic profile. This can be achieved by the use of the Phenomenex Kinetex C18 columns instead of other types of columns (e.g., ethylene bridged hybrid or Phenyl-Hexyl). Assessing an extensive steroidogenic profile greatly increases the mechanistic understanding of potential effects on steroidogenesis in COCs. This may be especially relevant when the model is used to evaluate EDCs that interact with steroidogenic pathways. However, our data show that targeted steroid measurements, e.g., using RIAs for progesterone and estradiol, may be equally predictive for these steroid hormones.

### 4.6 Effects of DES Exposure on Developmental Competence and Embryo Development

The nuclear and cytoplasmic maturation of oocytes, supported by the somatic cells of the follicle and secreted hormones, confers to the oocyte developmental competence. Upon fertilization, these oocytes will produce embryos. Exposure of oocytes to EDCs may reduce their developmental competence and the quality of embryos produced from them. For example, BPA-exposed bovine oocytes have been reported to yield less blastocysts upon fertilization compared to non-exposed oocytes (Choi et al., 2016). Fertilization of mono(2-ethylexyl)phthalate (MEHP)-exposed bovine oocytes results in blastocysts with altered transcriptomic and proteomic profiles (Kalo and Roth, 2015). However, the effects of EDCs on early embryo development has been less studied. Pre-implantation embryo development is considered a sensitive time-window in development, characterized by epigenetic reprogramming and perturbations of it might affect developmental gene expression (Ross and Sampaio, 2018). At a time when gene expression defines growth and early cell fate specification, EDC exposure might negatively influence embryo development. This developmental stage, prior to implantation, occurs along the oviduct. The fluid within the oviductal lumen consists a vital part of the developing embryos microenvironment. Both morphology of the oviduct and the composition of the oviductal fluid are influenced by steroid hormones such as estrogen and progesterone (Saint-Dizier et al., 2020). In light of that, EDCs, if their presence is verified within the oviduct, could influence the microenvironment of early embryos. Later stages of embryo development, e.g., post implantation, are also susceptible to the action of EDCs, as evidenced by the case of the uterine tract abnormalities observed in *in utero* DES-exposed women, believed to occur due to dysregulated expression of genes controlling the reproductive tract formation during embryo development (Reed and Fenton, 2013).

#### 4.6.1 Principle of Method

**FIGURE 10:**
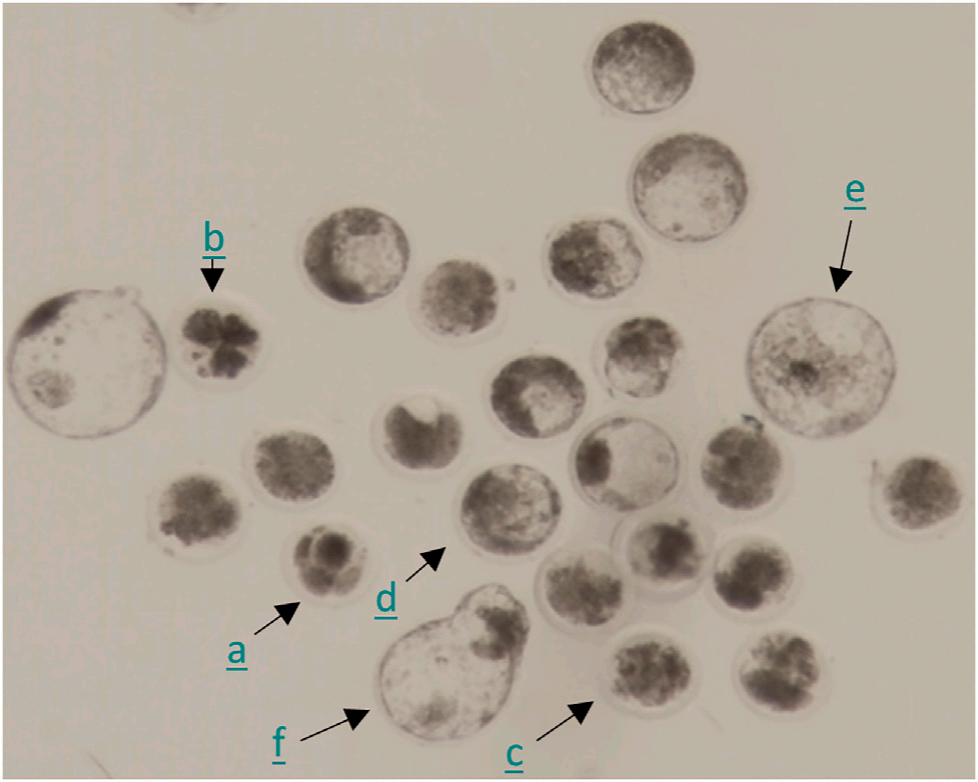
Representative photomicrograph of embryos 8 days post fertilization. Combination of embryos of which the development was arrested after the first few cleavages (a), fragmented embryos (b), morulas (c), and blastocysts of different developmental stages (early (d), expanded (e), hatching (f)).

To assess effects of DES on developmental competence acquisition, bovine oocytes were exposed to DES during IVM and subsequently fertilized in absence of DES. Next, the presumptive zygotes were cultured for 8 days *in vitro* (Figure 10). To examine whether DES may impact early embryo development, oocytes were matured and fertilized in the absence of DES, and the presumptive zygotes were exposed to DES during IVC (Days 1–8). In both cases, embryo cleavage was recorded on Day 5 and blastocyst number on Day 7 and Day 8. A representative image of embryos acquired 8 days after fertilization can be found on Figure 10. A comprehensive visual identification of bovine embryo developmental stages can be found in Wei et al. (2017) and Ushijima and Akiyama (2009).

#### 4.6.2 Results

**FIGURE 11:**
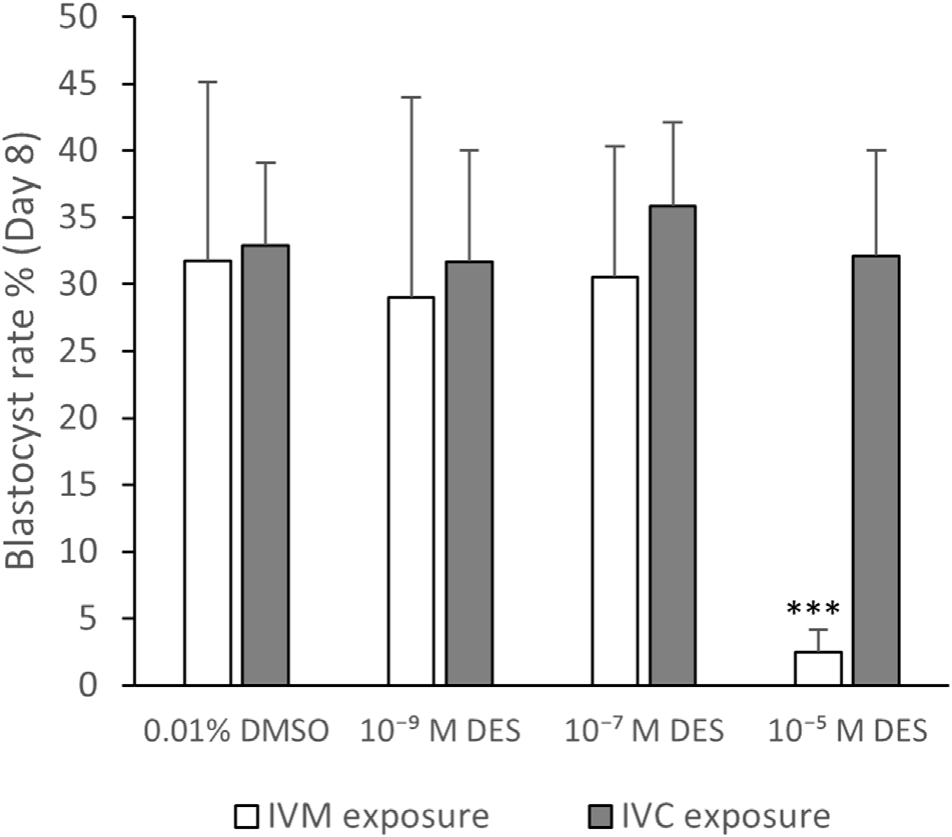
Blastocyst rate. White bars represent groups of embryos produced by fertilization of oocytes exposed to DES during in vitro maturation (IVM). Grey bars represent groups of embryos produced from routine oocyte IVM and IVF, and then embryo exposure to DES for 8 days of *in vitro* culture (IVC). A high DES concentration (10^−5^ M DES) statistically significantly reduced the developmental potential of oocytes, but exposed embryos were able to develop to blastocysts in normal rates. Bars represent mean of three replicates (±SD). ***Statistically significantly different from vehicle-treated control oocytes (*p* < 0.001) Number of fertilized oocytes analyzed per group, per independent experiment, and per condition (IVM or IVC exposure) are reported in Supplementary Table S1.

Historical data from our lab show that the success rate for blastocyst development is approximately 30%. Exposure to DES (10^–5^ M) during IVM resulted in a 3% blastocyst rate on Day 8 after fertilization, which is statistically significantly lower (*p* < 0.0001) compared to embryos produced from vehicle-treated control oocytes (32%) (Figure 11, white bars). No statistically significant effects were observed when embryos were produced from oocytes matured in media containing 10^–7^ M DES or 10^–9^ M DES with blastocyst rates of 29% and 30% respectively. Yet, cleavage rates were unaffected by DES exposure during IVM when compared to the vehicle-treated group as shown in Table 3.

**TABLE 3:** Cleavage rates of embryos produced after *in vitro* fertilization of oocytes exposed to different concentration of DES during *in vitro* maturation.

	Experimental group						
			Control	0.01% DMSO	10^–9^ M	10^–7^ M	10^–5^ M
Cleavage	Uncleaved	Count*	58	64	82	74	88
		%	21^a^	23^a,b^	29^a,b^	26^a,b^	32^a^
	≤8-cell	Count*	103	114	85	104	114
		%	37^a,b^	40^a^	30^b^	37^a,b^	41^a^
	>8-cell	Count*	116	104	116	103	73
		%	42^b^	37^a,b^	41^b^	37^a,b^	27^a^

*Total count of three independent experiments (Supplementary Table S1).

a,b Values in the same row without a common superscript letter are statistically significantly different (*p* < 0.05).

When embryos were exposed to DES, cleavage rates were similar between all DES-exposed and non-exposed groups (Table 4). Blastocysts rates were also not altered with DES exposure and were around 32% (Figure 11, grey bars). The embryos exposed to 10^–7^ M DES showed a blastocyst rate of 36%. Thus 10^–5^ M DES only affected blastocyst rates when it was added during IVM, prior to *in vitro* fertilization, while it had no effects on blastocyst rates when added post fertilization to presumptive zygotes (Figure 11).

**TABLE 4:** Cleavage rates of embryos exposed for 8 days to different concentrations of DES during *in vitro* embryo culture.

Experimental group							
			Control	0.01% DMSO	10^–9^ M	10^–7^ M	10^–5^ M
Cleavage	Uncleaved	Count*	47	45	43	50	31
		%	20^a^	19^a^	18^a^	21^a^	14^a^
	≤8-cell	Count*	106	99	97	86	102
		%	44^a^	41^a^	40^a^	36^a^	44^a^
	>8-cell	Count*	87	95	100	104	97
		%	36^a^	40^a^	42^a^	43^a^	42^a^

Total count of three independent experiments (Supplementary Table S1).

a Values within the same row with the same superscript letter are not significantly different.

#### 4.6.3 Technical Aspects and Limitations

Current strategies for the production of embryos, as the one presented here, have a low yield of blastocysts produced per fertilized oocytes. The protocol followed here has a 30–40% blastocyst rate, increasing thus the number of oocytes and consequently of ovaries needed for the production of embryos. Additionally, the timing chosen to record cleavage rates (i.e., Day 5) allows not only recording cleavage, but also the proportion of ≤8-cell to >8-cell embryos. This may provide an insight on potential interference with either cleavage early on in development (≤8-cell) or major embryo genome activation that occurs at the 8-to-16 cell stage in the bovine. Although cleavage and blastocyst data are an indication of developmental competence acquisition and embryo growth, when assessing effects of EDCs, a more comprehensive analysis of the blastocyst quality might be warranted to pinpoint small but significant perturbances within the embryo.

## 5 Discussion

Infertility is a public health priority estimated to affect around one in six couples worldwide. Underlying medical conditions and lifestyle factors, such as smoking, diet, and exercise, have been found to be causing or contributing factors (Chavarro et al., 2007; Wise et al., 2012; Radin et al., 2014; Heger et al., 2018; Aoun et al., 2021). Increasingly, studies have shown association between exposure to environmental contaminants and infertility, and among them is a particular class of chemicals that causes disturbances of the endocrine system, namely the endocrine disrupting chemicals (EDCs). There is evidence that these chemicals can interfere with normal function of both the male and female reproductive system, although data on the effects on female reproductive health is underrepresented (Krzastek et al., 2020; Fucic et al., 2021; Green et al., 2021). Studies have revealed a correlation between levels of specific EDCs in biological fluids (e.g., follicular fluid, or blood) of women and sub- or infertility (Akkina et al., 2004; Meeker et al., 2011; Ehrlich et al., 2012; Petro et al., 2012a). *In vitro* and *in vivo* experimental studies have shown effects of various EDCs on aspects of female reproductive health like ovarian weight, folliculogenesis and oocyte maturation (Chapin et al., 1999; Rodríguez et al., 2010; Manikkam et al., 2013; Hannon and Flaws, 2015b; Rattan et al., 2018; Ding et al., 2020). The latter results in the acquisition of competence by the oocyte to be fertilized successfully and subsequently develop to a blastocyst. Here, we describe several key biological processes involved in oocyte maturation that can be measured *in vitro* using bovine oocytes. To determine the applicability and predictivity of the bovine model for female reproductive toxicity testing and EDC identification, the bovine oocytes were exposed to the known human EDC diethylstilbestrol (DES) during *in vitro* maturation, or fertilized oocytes were exposed during *in vitro* embryo culture.

### 5.1 The *In Vitro* Bovine Oocyte Model

A great part of available data in reproductive toxicology originates from studies, *in vivo* or *in vitro*, in rodent models. The collection of oocytes, embryos or any biologically relevant material for these studies requires the sacrifice of animals. Substituting *in vitro* assays in these models with the bovine model, eliminates the need for additional animal sacrifice, as cow ovaries can be easily obtained from a local slaughterhouse, providing a relatively high number of oocytes per animal (10–20). Moreover, the endpoints discussed here, such as cumulus expansion quantification, nuclear maturation, and steroid hormone quantification, can be easily combined to be analyzed in the same biological material. The benefits of the model, however, extend beyond compliance with the 3R principle in animal use in testing. There are shared similarities between the human and bovine species, including, but not restricted to, mono-ovulation, oocyte diameter, oocyte transcriptome, time to 2-cell stage embryo upon fertilization, and the timing of oocyte maturation and early embryo development (Ménézo and Hérubel, 2002; Santos et al., 2014). The usefulness of this model for the human situation has been previously discussed in (Santos et al., 2014). Additionally, the validity and predictability of the model, specifically of nuclear maturation in the bovine oocyte, has been confirmed for reproductive toxicology screening by various studies, for example in the ReProTect project (Lazzari et al., 2008; Luciano et al., 2010; Beker van Woudenberg et al., 2012).

It is important to consider that bovine ovaries obtained post-mortem from slaughterhouse cows are not of a standard genetic origin and that this situation differs from experimental animals where specific breeding lines are used. A consequence of this approach is that the ovaries collected are of unknown origin with regards to the age, health status, estrus cycle stage, and dietary profile of the cows. Moreover, oocytes isolated from the ovaries of different animals are pooled together within experimental groups. Additionally, previous exposure of the slaughtered cows to EDCs is unknown. Therefore, data may present with increased variability that is difficult to attribute to aforementioned factors. Compared to rodent models, the bovine resembles to a higher extent human reproduction when addressing final oocyte maturation and early embryo development. However, *in vivo* testing in rodent models is undeniably useful since multiple modes of action and feedback mechanisms leading to multiple systemic interactions elicited by EDCs and their metabolites cannot easily be recapitulated *in vitro*. The six key biological processes we describe here though, can be assayed on the bovine *in vitro* model of oocyte maturation and developmental competence acquisition.

### 5.2 The Applicability for EDC Identification

DES is a synthetic non-steroidal estrogen that has previously been shown to cause failure of oocyte nuclear maturation in the mouse model of *in vitro* oocyte maturation. The effective exposure levels (in the range of 20–60 µM DES) were higher than the DES concentrations used in this study. Data indicated that failure to reach the MII-stage was reversible and the observed aberrations were due to meiotic spindle assembly and chromosome alignment defects (Ding et al., 2020). Our data show that only very high concentrations of DES were able to illicit statistically significant effects in *in vitro* matured bovine oocytes. Oocyte nuclear maturation was hindered at 10^–5^ M DES, with only a small fraction of the oocytes reaching the MII stage. The mechanism leading to this could possibly be similar to the one described after DES exposure of mouse oocytes, pertaining failure of meiotic spindle assembly. Oocytes exposed to lower concentrations of DES were able to normally resume and progress meiosis to the MII stage, although the ploidy status of these oocytes was not determined in this study. DES and other EDCs have been shown to have aneugenic potential making oocyte aneuploidy incidence a prominent endpoint that could be included in the future (Kayani and Parry, 2008).

Nuclear maturation is concurrent with cytoplasmic maturation in the making of a developmentally competent oocyte. As a part of cytoplasmic maturation, we examined mitochondrial (re)distribution, which normally occurs during bovine oocyte maturation from a peripheral to a centrally diffused pattern, and primarily depends on the cytoskeleton (Ferreira et al., 2009). Excessively clustered or localized mitochondria in an oocyte might lead, after fertilization, to blastomeres of the embryo inheriting less than optimum amount of mitochondria. The distribution of active mitochondria in bovine oocytes was not affected by the presence of DES at any of the concentrations tested. Important roles of mitochondria that could be further studied for their sensitivity to endocrine action include ATP production, Ca^2+^ signaling and lipid metabolism. Mitochondria and mtDNA copy number are also candidates for the assay, as they normally increase during oocyte maturation to ensure a sufficient mitochondrial complement able to support early embryo development after fertilization (Ferreira et al., 2009). Although GVBD signals the arrest of transcription of genomic DNA in the oocyte, mtDNA continues being transcribed and can be regulated by estradiol through ERα and ERβ (Klinge, 2020). Therefore, it could be postulated that exposure to DES might affect mitochondrial function by altering the expression of the enzymes involved in oxidative phosphorylation. Besides mitochondria, other aspects of cytoplasmic maturation that could serve as sensitive endpoints are the redistribution of other cytoplasmic organelles (e.g., endoplasmic reticulum), the formation of the male pronucleus upon fertilization, cortical granule exocytosis, and maternal factors.

The cumulus investment is an essential component of the COC that shares a bidirectional relationship with the oocyte and dictates synchronous follicle-oocyte growth, ovulation, and fertilization (Tanghe et al., 2003; Chen et al., 1993). In the late stages of oocyte maturation, cumulus cell (CC) expansion occurs through the synthesis of a hyaluronic acid rich extracellular matrix and cell movement that disrupts connections among cumulus cells (Kawashima et al., 2012). The expansion is dependent both on factors secreted by LH-stimulated granulosa cells and the oocyte (Park et al., 2004). In FSH-superstimulated heifers, CC expansion has been associated with increased developmental competence of *in vivo* matured oocytes (Aardema et al., 2013b). Here, exposure of bovine oocytes to 10^–9^ M and 10^–7^ M DES during IVM did not affect the fold-increase of the projected surface area of COCs, but there was a significantly smaller expansion after exposure to the highest DES concentration tested (10^–5^ M). Although high DES concentrations inhibited oocyte nuclear maturation and COC expansion, there was no increase of apoptotic events and necrotic rates were only minimally increased. When steroid levels were quantified in maturation media, DES was found to increase the production and/or secretion of progesterone and pregnenolone. This effect could be an indication of enhanced luteinization occurring in the cumulus, driven possibly by altered steroidogenic enzyme activities early in the steroidogenic pathway (e.g., StAR, CYP11A1, 3βHSD). Cumulatively, the effect of high DES exposure on the cumulus-oocyte complex appears to be through specific mechanisms and not a general induction of apoptosis.

Developmental competence, observed as blastocyst number per fertilized oocytes, was also affected by the highest concentration of DES, as was expected from oocytes not having completed meiosis. Interestingly, the percentage of oocytes in MII exposed to 10^–5^ M DES (3%) is equal to the blastocyst rate (3%) of oocytes of the same group. It is possible that the fittest oocytes that do complete meiosis upon DES exposure and respond or adjust to such perturbations, are also the most developmentally competent oocytes and are resistant to the action of this ER agonist. Intriguingly, none of the DES exposed oocyte groups showed differences in cleavage rates post-fertilization. Embryo cleavage is dependent on cytoplasmic maturation and accumulation of maternal transcripts, suggesting that the factors needed to initiate and complete cleavage have been accumulated normally during maturation, although possibly during earlier stages of oocyte growth (preantral) than studied here (Memili and First, 1998). Cleavage rates were also recorded as normal after fertilization of oocytes exposed to a high concentration of DES (shown not to complete meiosis), however it is likely that these are events of cytoplasmic fragmentation resulting in anucleate blastomeres. When exposure to DES was restricted to the IVC procedure of embryo culture, and not during *in vitro* maturation of the oocytes, no effect was observed in blastocyst rates. This denotes the differential susceptibility of an oocyte and an early embryo to endocrine action, although further investigation of the quality of the blastocysts produced is needed. If failure to complete meiosis was indeed a product of spindle assembly abnormalities, it could be possible that the insensitivity of embryos to the presence of DES is due to differences in the organization and dynamics of the meiotic and mitotic spindle. Though, to firmly establish such an insensitivity, follow-up analysis on blastocysts could include data on allocation of cells to the inner cell mass or trophectoderm lineage, total cell count, ploidy status, and transcriptomic profiling.

**FIGURE 12:**
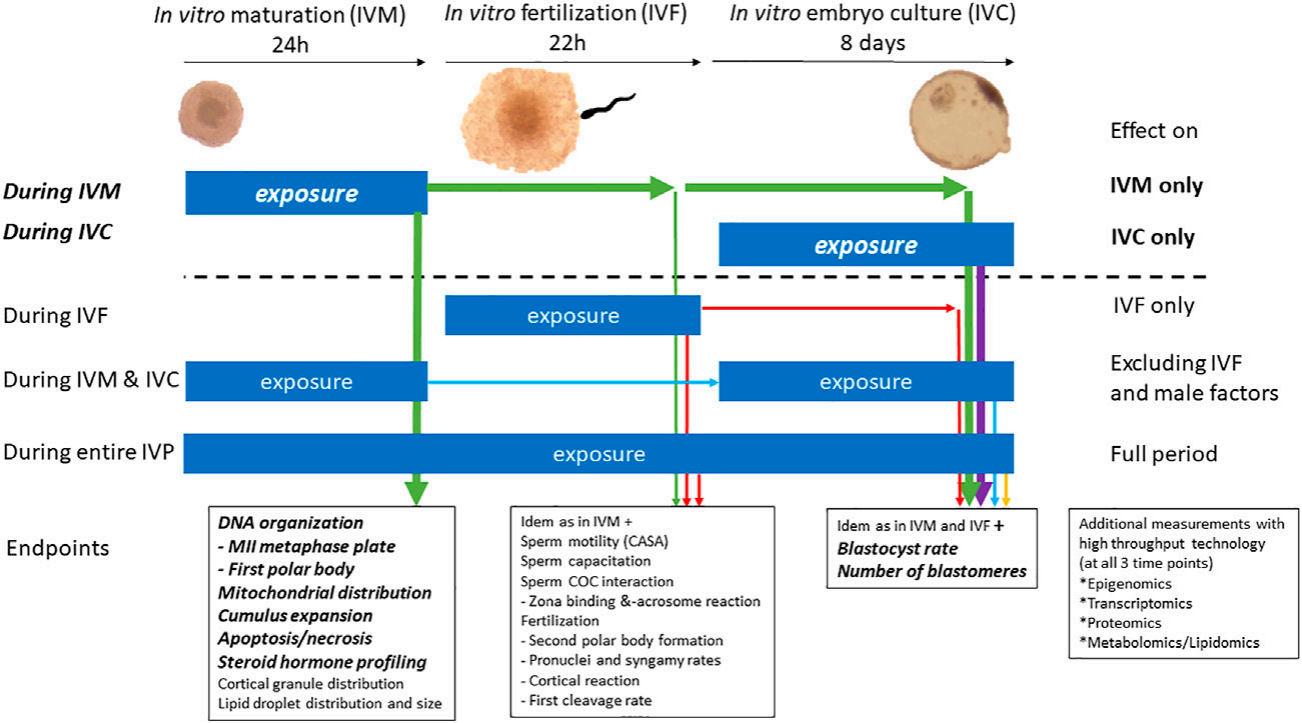
Potential applications of the bovine model of *in vitro* embryo production in reproductive toxicology for the identification of EDCs. The bold and italic text above the dotted line refer to experiments reported herein, namely exposure during *in vitro* oocyte maturation or during *in vitro* embryo culture. Exposures under the dotted line indicate extended possibilities with the herein described *in vitro* bovine oocyte and embryo model.

The proposed assay revealed that COC expansion, steroidogenesis, nuclear maturation, and developmental competence, all important aspects of oocyte maturation, are affected by DES. However, statistically significant effects were observed only when oocytes were exposed to the highest DES concentration. The binding of ERs by DES represents a common but very specific mechanism of EDC action, and in our ongoing research we aim to include compounds covering a broader spectrum of mechanisms, like the chemical ketoconazole which is known to inhibit the activity of steroidogenic enzymes of the CYP450 family. This may further corroborate the applicability of this model in identifying EDCs that induce reproductive toxicity through interference with oocyte maturation or embryo development. A graphical summary of potential exposure experiments and relevant endpoints, using the *in vitro* bovine model for oocyte maturation, fertilization and early embryo culture, is presented in Figure 12. EDCs under investigation can be used for exposure experiments in various stages of the *in vitro* embryo production protocol, as has been indicated in Figure 12. After exposure, the oocytes and embryos developmental capacities can be followed. The IVP protocol described here provides a useful tool for toxicological studies and makes oocytes, zygotes and embryos easily accessible whilst adhering to the 3R principle. However, there are limitations to the insights this model can provide and we have listed for each of our endpoints some considerations to take into account for interpretation of the experimental data. Moreover, the temporal window of either oocyte maturation or embryo development that can be studied through *in vitro* procedures in the bovine, in itself important, reflects a small portion of *in vivo* processes in the development of an oocyte or an embryo. Females can be exposed *in utero* to EDCs and continue being exposed through childhood, puberty, and adulthood. For example, germ cell specification, early-to-late folliculogenesis, follicle pool activation, follicle dominance, and ovulation are grossly overlooked at the moment in comprehensive studies. Beyond these restrictions, the developed endpoints in the bovine *in vitro* oocyte maturation, fertilization and embryo development model, with the use of fresh ovaries obtained from slaughterhouses, allow to work experimental animal free and provide valuable insights in the direct effects of EDCs on cumulus cells, oocytes and embryos as well as sperm cells (outside the scope of this manuscript). It is important that currently available and newly developed tools are implemented in (regulatory) chemical safety assessment processes to address shortfalls in the current EDC testing strategies, especially when it comes to assessment of female reproductive toxicity.

## Data Availability

The original contributions presented in the study are included in the article/Supplementary Material, further inquiries can be directed to the corresponding author.
