# Supplementary material for: Bovine In Vitro Oocyte Maturation and Embryo Production Used as a Model for Testing Endocrine Disrupting Chemicals Eliciting Female Reproductive Toxicity With Diethylstilbestrol as a Showcase Compound
Source: Front Toxicol. 2022 May 24;4:811285. doi: 10.3389/ftox.2022.811285 (PMC9171015; doi:10.3389/ftox.2022.811285)
Supplement: Supplementary file 1 [file Table1.docx]

**Supplementary table S1**: Number of COCs/oocytes (^1^) or cumulus cells (^2^) or fertilized oocytes (^*^ and ^#^) analyzed per experimental replicate per endpoint analyzed.

| Endpoint | Independent experiment | Control | 0.01% DMSO | 10^-9^ M DES | 10^-7^ M DES | 10^-5^ M DES |
| --- | --- | --- | --- | --- | --- | --- |
| Nuclear maturation | Exp. 1^1^ | 28 | 32 | 28 | 28 | 27 |
|  | Exp. 2^1^ | 32 | 29 | 27 | 37 | 36 |
|  | Exp. 3^1^ | 30 | 29 | 33 | 36 | 34 |
| Mitochondrial distribution | Exp. 1^1^ | 9 | 8 | 9 | 7 | 15 |
|  | Exp. 2^1^ | 18 | 8 | 8 | 7 | 14 |
|  | Exp. 3^1^ | 21 | 20 | 17 | 19 | 19 |
|  | Exp. 4^1^ | 13 | 21 | 17 | 18 | 24 |
| COC expansion | Exp. 1^1^ | 33 | 34 | 32 | 34 | 34 |
|  | Exp. 2^1^ | 34 | 27 | 32 | 34 | 34 |
|  | Exp. 3^1^ | 33 | 32 | 34 | 31 | 34 |
| Apoptosis/Necrosis quantification | Exp. 1^1,2^ | - | n^1^: 12  n^2^ : 5356 | n^1^: 12  n^2^ : 7391 | n^1^: 12  n^2^ : 6742 | n^1^: 13  n^2^ : 9235 |
|  | Exp. 2^1,2^ | - | n^1^: 12  n^2^ : 7148 | n^1^: 12  n^2^ : 9984 | n^1^: 12  n^2^ : 7124 | n^1^: 13  n^2^ : 9425 |
|  | Exp. 3^1,2^ | - | n^1^: 12  n^2^ : 5012 | n^1^: 12  n^2^ :5843 | n^1^: 12  n^2^ : 7879 | n^1^: 11  n^2^ : 10037 |
| Cleavage and Blastocyst rate^*^ | Exp. 1^*^ | 94 | 96 | 100 | 96 | 94 |
|  | Exp. 2^*^ | 97 | 97 | 97 | 96 | 93 |
|  | Exp. 3^*^ | 86 | 90 | 86 | 89 | 88 |
| Cleavage and blastocyst rate^#^ | Exp. 1^#^ | 80 | 80 | 80 | 80 | 80 |
|  | Exp. 2^#^ | 80 | 79 | 80 | 80 | 70 |
|  | Exp. 3^#^ | 80 | 80 | 80 | 80 | 80 |

^1^ n-number of COCs

^2^ n-number of cumulus cells

^*^ Embryos were produced from oocytes exposed to DES during IVM only

^#^ Embryos were exposed to DES during IVC only, oocytes were not exposed to DES during maturation or fertilization
