# Supplementary material for: Bovine In Vitro Oocyte Maturation and Embryo Production Used as a Model for Testing Endocrine Disrupting Chemicals Eliciting Female Reproductive Toxicity With Diethylstilbestrol as a Showcase Compound
Source: Front Toxicol. 2022 May 24;4:811285. doi: 10.3389/ftox.2022.811285 (PMC9171015; doi:10.3389/ftox.2022.811285)
Supplement: Supplementary file 2 [file Table2.DOCX]

**SUPPLEMENTARY MATERIAL**

**Rinsing Medium R**

Rinsing medium R is composed as detailed in the table below in a final MilliQ water volume of 1 L, pH is measured and modified to 7.3 ± 0.05 and osmolarity is set to 280 ± 2 mOsmol. The medium is filtered (0.22 um) and stored in aliquots at -20^o^ C.

| Components (Medium R) |  |  |
| --- | --- | --- |
| NaCl | Merck 1.06404.1000 | 6.66 g |
| KCl | Merck 4936 | 0.235 g |
| NaHCO_3_ | Sigma S 4019 | 0.168 g |
| Na_2_HPO_4_ | Merck 6559 | 0.047 g |
| Sodium lactate (60%) | Sigma L 7900 | 2.17 mL |
| HEPES | Sigma H 147-100g | 2.38 g |
| Phenol red | Sigma P 0290 | 2 ml |
| CaCl_2_.2H_2_O | Merck 2382 | 0.39 g |
| MgCl_2_.6H_2_O | Merck 5833 | 0.10 g |
| Sodium pyruvate | Sigma P 2256 | 0.11 g |
| Penicillin/streptomycin | Gibco 15140-148 | 10 mL |
| Bovine serum albumin, Fraction V (BSA-FV) | MP Biomed. 81003 | 6 g |

**Medium D**

Medium D 10X (D10X) is necessary for the dilution of Percoll® to 90% and a 10x dilution (medium D1X) of it is used for the making of 45% Percoll® solution and for the making of sperm media.

Medium D10X is composed as detailed in the table below, pH is adjusted to 7.35 ± 0.05, osmolarity to 280±2 mOsmol, and filtered (0.22 um) for sterilization. Aliquots are stored at 4^o^ C. Medium D10X is diluted 10 times with LAL Reagent water and supplemented with 2% v/v phenol red to produce medium D1X.

| Component (D10X) |  |  |
| --- | --- | --- |
| LAL reagent water | Cambrex W50-500 | 100 mL |
| NaCl | Merck 1.06404.1000 | 5.4 g |
| KCl | Merck 4936 | 0.23 g |
| NaHCO_3_ | Merck 1.06329.0500 | 2.1 g |
| Na_2_HPO_4_ | Merck 6559 | 0.047 g |
| Sodium lactate (60%) | Sigma L 7900 | 4.29 mL |
| HEPES | Sigma H 6147-100g | 2.38 g |
| MgCl_2_.6H_2_O | Merck 5833 | 0.31 g |

**Percoll® solutions**

For a 90% solution, Percoll® is diluted in a 1:9 ratio with medium D 10X. Percoll 45% solution is made by a 1:1 dilution of Percoll® 90% with medium D 1X.

**Sperm medium**

Sperm medium composition is detailed below, and the medium is sterilized by filtration and stored at -20^o^ C.

| Components (sperm medium) |  |  |
| --- | --- | --- |
| Medium D 1X |  | 10 mL |
| Sodium pyruvate | Sigma P 2256 | 1.1 mg |
| Penicillin/streptomycin | Gibco 15140-148 | 0.1 mL |
| Bovine serum albumin fraction V | MP Biomed. 81003 | 0.06 g |

**Fertilization medium**

Fertilisation medium components are listed below, the medium is corrected for pH (7.3 ± 0.05) and osmolarity (280±2 mOsmol), sterilized by filtration and stored at -20^o^ C.

| LAL reagent water | Cambrex W50-500 | 0.2 L |
| --- | --- | --- |
| NaCl | Merck 1.06404.1000 | 1.26 g |
| KCl | Merck 4936 | 0.046 g |
| NaHCO_3_ | Sigma S 4019 | 0.42 g |
| Na_2_HPO_4_ | Merck 6559 | 0.0094 g |
| Sodium pyruvate | Sigma P 2256 | 0.00484 g |
| Phenol red | Sigma P 0290 | 0.400 mL |
| CaCl_2_.2H_2_O | Merck 2382 | 0.078 g |
| MgCl_2_.6H_2_O | Merck 5833 | 0.02 g |
| Penicilin/streptomycin | Gibco 15140-148 | 2 mL |
| BSA, fatty acid free | Sigma A6003 | 1.2 g |

**PHE solution**

All components, unless stated otherwise, were purchased by Sigma-Aldrich, St. Louis, Missouri, USA. The PHE solution has a composition of 25% v/v penicillamine 2 mM, 25% v/v hypotaurine 1 mM, and 1% v/v epinephrine 2.5 mM, in 0.9% NaCl. Hypotaurine (H1384) and D-penicillamine (P4875) are dissolved in 0.9 % NaCl. Epinephrine (E1635) is dissolved in sterile solution A (table below, pH 4) and kept protected from light. All solutions are stored at -20^o^ C.

| Components (Solution A) |  |  |
| --- | --- | --- |
| LAL Reagent water | Cambrex W50-500 | 50 mL |
| 60% Sodium lactate | Sigma L 7900 | 1.26 mL |
| Sodium metabisulfite | Sigma S-900 | 0.5 g |

**Synthetic oviductal fluid (SOF) media**

SOF media for embryo culture is composed of a 1:4 ratio of SOF A and SOF B media (composition of each below). The pH of SOF A and SOF B are adjusted to 7.3 ± 0.05 and the osmolarity of SOF A is adjusted to 300 ± 2 mOsmol. The components are sterilized by filtration and stored at 4^o^ C.

| Components (SOF A) |  |  |
| --- | --- | --- |
| LAL reagent water | Cambrex W50-500 | 200 mL |
| NaCl | Sigma S 5886 | 1.5725 g |
| KCl | Sigma P 5405 | 0.1335 g |
| KH_2_PO_4_ | Sigma P 5655 | 0.0405 g |
| Sodium lactate (60%) | Sigma L 7900 | 150 µL |
| MgSO_4_.7H_2_O | Merck 1.05886.500 | 0.0455 g |
| NaHCO_3_ | Sigma S 4019 | 0.525 g |
| CaCl_2_.2H_2_O | Sigma C 7902 | 0.0655 g |
| Phenol red | Sigma P 0290 | 125 µL |
| MEM non-essential Amino Acid Solution (100×) | Sigma M 7145 | 2.5 mL |
| BME amino Acid Solution (50×) | Sigma B 6766 | 5 mL |

| Components (SOF B) |  |  |
| --- | --- | --- |
| LAL reagent water | Cambrex W50-500 | 20 mL |
| Penicillin/Streptomycin | Gibco 15140 | 100 µL |
| Sodium Pyruvate | Sigma P 2256 | 3.6 mg***** |
| L-Glutamine | Sigma G 8540 | 0.030 g |
| Bovine serum albumin | Celliance 81-001-4 | 0.40 g |
