# Supplementary material for: Bovine In Vitro Oocyte Maturation and Embryo Production Used as a Model for Testing Endocrine Disrupting Chemicals Eliciting Female Reproductive Toxicity With Diethylstilbestrol as a Showcase Compound
Source: Front Toxicol. 2022 May 24;4:811285. doi: 10.3389/ftox.2022.811285 (PMC9171015; doi:10.3389/ftox.2022.811285)
Supplement: Supplementary file 3 [file DataSheet1.docx]

**Supplementary Figure 1:** Quantification of progesterone (P2) and estrogen (E2) in media of COC cultures using a radioimmunoassay (RIA).


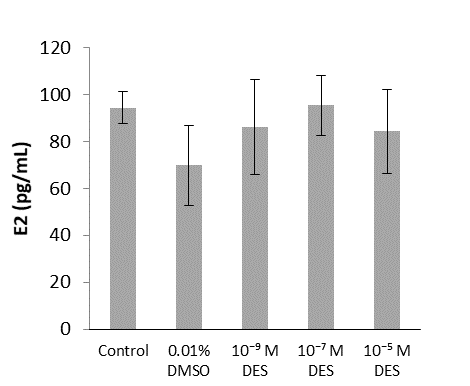

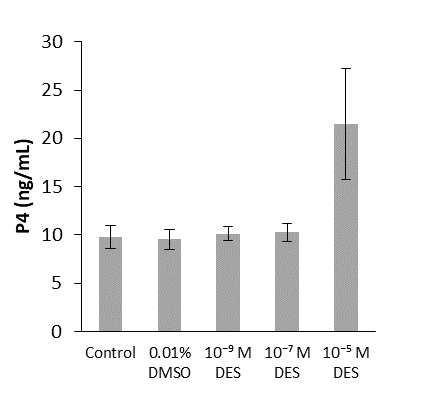


***

Effect of DES on select steroids produced by bovine cumulus cells in an in vitro system of COC maturation. Progesterone (P4) and 17β-estradiol (E2) levels, in medium in which COCs were in vitro matured in for 24 hours, were quantified by RIA assay (detailed below). P4 levels in the medium were significantly higher when COCs were treated with 10^-5^ M DES, compared to the vehicle-treated group (*p* < 0.001)

Assay:

The concentrations of progesterone and 17β-estradiol were determined in aliquots of 100 μL medium. Oocytes in groups of 70 are matured in 500 μL of maturation media, with or without DES, and the conditioned media is collected at the end of IVM, snap frozen in liquid nitrogen and thawed right before analysis. The samples were analyzed using solid-phase[125I] RIA (IM1188, Beckmann Coulter, California, USA; progesterone, RIA-4381 CT, DRG Diagnostics GmbH, Marburg, Germany; 17β-estradiol) according to the manufacturer with slight modifications. Quantification of 17β-estradiol in the sample required an extraction step. Briefly, cell culture media were extracted with 2 mL diethyl ether (Scharlau, Barcelona, Spain) and an internal standard of 167 Bq [3H] steroid was added before extraction in order to determine and correct for the efficiency of extraction. After evaporation of the organic solvent, the samples and efficiency series were dissolved in 125 mL borate buffer. Duplicate volumes of the samples, for both 17β-estradiol and progesterone quantification, were incubated in antibody-coated tubes. Calculation of hormone concentrations was performed by applying the approximation for the standard series from RIA Smart (Packard Instruments Company, Meriden, CT, USA). The concentrations calculated using RIA differed <4% from the defined concentrations of the standards used to build the reference curves. The intra-and inter assay coefficients of variation were <10% for all assay (modified from Aardema et al., 2013).
